# The Geography of Frail Ageing in Italy: Longevity, Non-Self-Sufficiency and Implications for Assistance Benefits

**DOI:** 10.3390/ijerph23080947

**Published:** 2026-07-23

**Authors:** Carlo Maccheroni, Roberta Pace, Giuseppe Venere

**Affiliations:** 1 Department of Economics, Social Studies, Applied Mathematics and Statistics, University of Turin, 10153 Turin, Italy; carlo.maccheroni@unito.it; 2 Department of Political Science, University of Bari Aldo Moro, 70121 Bari, Italy; giuseppe.venere@uniba.it

**Keywords:** healthy life expectancy, longevity inequalities, functional limitations, territorial disparities, gender gap in ageing, Italy

## Abstract

**Highlights:**

**Public health relevance—How does this work relate to a public health issue?**

- Italy’s exceptional longevity conceals strong territorial and gender inequalities in frailty, functional limitations and non-self-sufficiency.
- Mapping the geography of frail ageing helps identify regions where older adults face higher vulnerability and greater pressure on health and social care systems.

**Public health significance—Why is this work of significance to public health?**

- This study links the extension of life to the quality of survival, showing that longer life often coexists with disability, multimorbidity and dependency.
- It highlights persistent north–south disparities in healthy ageing, revealing unequal regimes of later-life health and welfare needs across Italian regions.

**Public health implications—Key implications for practitioners, policy makers and researchers.**

- Territorial differences in frailty and assistance needs call for region-specific long-term care strategies, especially in southern Italy and among older women.
- Integrating demographic, health and welfare indicators can support more equitable resource allocation and strengthen the effectiveness of ageing-related public policies.

**Abstract:**

This paper examines the geography of frail ageing in Italy by analysing how longevity, health conditions, non-self-sufficiency and assistance needs intersect across territories. Its distinctive contribution lies in moving beyond a purely demographic reading of population ageing, linking the extension of life to the quality of later-life survival and to the uneven territorial distribution of public assistance. Drawing on official data from Istat, Eurostat and INPS, this study adopts a regional comparative approach, with evidence disaggregated by sex, age group and macro-area. The analysis combines indicators of demographic structure, residual life expectancy at age 65, healthy life expectancy, severe limitations in daily activities, multimorbidity and assistance benefits. This framework makes it possible to distinguish between ageing as a numerical increase in the older population and ageing as a differentiated condition of vulnerability, dependency and welfare need. The findings show that Italy’s high longevity conceals marked territorial and gender inequalities. While the centre–north displays more favourable survival and health profiles, the south and the islands have higher levels of frailty, functional limitations and reliance on public assistance. Women appear to be particularly exposed to this imbalance, living longer but spending more years in conditions of disability and dependency.

## 1. Introduction

Among the 27 countries of the European Union (EU), Italy has stood out for many years as the “oldest” country in Europe—followed at a short distance by Portugal, Bulgaria and Greece [1]—with the highest proportion of older people in the population. Older people are individuals aged over 65, an age group which, in the practice introduced by gerontology, can be divided into three subsets: young old (65–74), old (75–84) and oldest old (85 and over) [2]; these subsets generally mark the stages in the progressive deterioration of health conditions among older people. Population ageing is by now a long-term characteristic in Italy, having begun at the start of the 1980s as a result of both the persistent decline in fertility, with the consequent continuous decline in births and therefore in the number of young people, and the progressive reduction in mortality at pre-senescent ages [3], which has led Italy to rank among the most long-lived countries. This process has resulted in life duration becoming increasingly less differentiated within the population, to the extent that current mortality tables project a 90% probability for males and a 94% probability for females of surviving up to age 65 for the cohorts born in recent years.

The now consolidated extension of life, while highlighting the extraordinary successes achieved by medicine and personal care, is nevertheless shaping Italy into an ageing society because of the considerable weight that older people have in the demographic structure—22.3% in the case of men and 27.0% in the case of women at the beginning of 2025—with inevitable repercussions on the sustainability of the welfare system. Given that the growth of the older population has been accompanied by a diversification in the demand for care and health services, the demographic segment that has experienced the greatest growth over the last four decades is precisely those over the age of 80, in which the female component clearly prevails. This is also the age group that exerts the greatest pressure on health services because it is more vulnerable in terms of health conditions, as it is more exposed to the risk of chronic degenerative diseases, loss of autonomy and therefore non-self-sufficiency. As we will discuss later, gender differences are even more accentuated in these areas, to the disadvantage of the female component. Overall, the consequence is also greater pressure on the sector of personal assistance services.

Support roles that were once largely carried out by families for the protection and support of older people are slowly being transferred to the state and fulfilled through expenditure on assistance, reaching EUR 164.4 billion in 2023 (equal to approximately 7.7% of the GDP), which has always been borne by general taxation [4]. However, despite these significant financial commitments, the substantial gender differences in health conditions among older people, as well as the persistence of a north–south socio–health gradient (to the disadvantage of the south), continue to interact in different ways with access to and the provision of welfare services. Territorial conditions that have accumulated over time within the social protection system therefore remain, and, as a result, so have the conditions of hardship that continue to characterise the regions of southern Italy [5]. What causes the welfare system to be under pressure is not only the increase in the number of older people but also the composition of this population from a health and functional point of view.

The following analysis explores the core features of demographic ageing and contextualises them within regional differences in the health status of the older population in Italy while also considering that at the beginning of 2025, almost one quarter of pensions were assistance-based in nature [6] and intended to support pensioners in precarious health conditions and/or situations of economic hardship. Against this background, this article addresses two main research questions: First (RQ.1), how are the demographic and survival conditions of the older population territorially distributed across Italy, and what differences emerge among the various areas of the country in terms of longevity, frailty and quality of life? Second (RQ.2), what differences can be identified between north, central and southern Italy in terms of the distribution and intensity of assistance benefits directed to the older population?

## 2. Literature Review

Life expectancy has long occupied a central place in demographic research because it condenses the overall level of mortality in a population into a single indicator. Its importance lies in the fact that it cumulatively reflects the configuration of mortality risks across the life course, thereby providing a privileged measure for describing survival regimes and their transformation over time. In low-mortality countries, the long-term trend in longevity has shown a remarkably regular increase, which challenges the idea of a fixed and imminent limit to the expansion of human life and has shifted scholarly attention away from biological thresholds towards the social, health-related and institutional mechanisms that make longer survival possible [7]. At the same time, the literature has made clear that, while indispensable, life expectancy does not fully capture the interpretive complexity of longevity. While it may provide information about the quantity of life, it does not address the qualitative distribution of years lived or the health conditions, levels of autonomy or care needs that accompany longer survival [8,9].

From this perspective, contemporary scholarship on ageing has progressively moved beyond a purely quantitative reading towards an approach that links length of life to quality of survival [10]. Longer life does not automatically mean that the additional years gained are lived in good health, free of functional limitations or under conditions of full autonomy. It is precisely this awareness that has led to the growing use of indicators such as healthy life expectancy, disability-free life expectancy and, more broadly, health expectancies, all of which combine mortality and morbidity within a single analytical framework [11,12]. Within this perspective, the key question is no longer simply how long people live, but how many of those years are actually lived without disability, multimorbidity or the need for continuous care [13]. The literature has thus increasingly shown that longer survival may coexist with persistent or even rising exposure to frailty, which makes any linear interpretation of ageing as a simple “success” of modern health improvement inadequate [14].

This framework is particularly relevant in the Italian case. Italy is consistently one of the countries in Europe that has the highest human longevity, but this demographic primacy coexists with a geography of mortality and survival that is far from uniform [8,15]. Demographic research has long shown that territory is not a neutral background but rather a constitutive dimension of health and mortality processes. Provincial and regional analyses carried out in the early 2000s already highlighted how mortality differentials in Italy reflected the combined effects of social, economic, environmental and healthcare factors, thereby underscoring the role of local contexts in generating survival inequalities [16,17]. A spatial reading of mortality levels has also made it possible to move beyond overly simplified representations of Italy’s territorial divide, showing that, while remaining a structural axis of national demographic heterogeneity, the north–south gap does not take the same form at all ages, for both sexes or across all causes of death [13,18].

More recent research has confirmed that these differences are not merely a legacy of the past. Long-term regional analyses indicate that although life expectancy has increased overall, the convergence across Italian regions has progressively weakened, raising the possibility that it has effectively ceased. Before the pandemic, life expectancy was, on average, higher in the centre–north than in the south, with a fairly clear territorial gradient, especially among men [19]. Recent local-level studies have also found a slight increase in the territorial variability in mortality over the first two decades of the century, which, although not dramatic in absolute terms, included a growing component of between-region differentiation and a persistent pattern of within-region inequality [20]. This is compounded by evidence that specific components of mortality, such as seasonal mortality, contribute non-negligibly to widening territorial inequalities, particularly to the disadvantage of southern and island regions, thereby reinforcing the idea of a selective geographical vulnerability in Italian survival patterns [21].

When attention shifts from the duration of life to its quality, the Italian territorial divide becomes even more pronounced [19]. Studies on disability-free life expectancy have shown that although Italy records very high levels of life expectancy at age 65, the picture becomes less favourable once disability is taken into account [22,23]. The gap between the quantity and quality of survival is particularly relevant at older ages and is unequally distributed along the lines of gender, education and territory. Regional analyses have shown that disability-free life expectancy at age 65 varies considerably across areas of the country and across social groups, which indicates that additional years of life are not equally distributed in terms of functional autonomy. The disadvantage of southern Italy clearly emerges from this perspective as well, as does the role of low educational attainment, which significantly reduces the number of years lived free from disability [15]. In this sense, later-life survival cannot be interpreted solely as the outcome of an advanced demographic transition; rather, it must also be understood as a product of resources and disadvantages that are unevenly distributed across Italian territories [24].

Quality of survival depends not only on the presence or absence of disability, but also on the growing prevalence of multimorbidity and frailty [16]. In Italy, multimorbidity has become an increasingly structural feature of older age that rises rapidly with advancing age, affecting demand for healthcare services, home care and continuous assistance [25]. This dimension is not territorially neutral. On the one hand, studies conducted in different regional settings have identified higher levels of frailty in southern Italy than in northern areas, which suggests that biological and social ageing do not unfold everywhere with the same intensity [26]. On the other hand, the experiences of frail older adults reveal particularly clearly the combined impact of chronic illness, functional limitations and barriers to accessing services, such as waiting lists, out-of-pocket costs, shortages of home-based social and healthcare services, and insufficient integration between health and social care. Together, these factors can turn prolonged survival into a condition of greater fragility and care deprivation, thus exposing older people to higher risks or inducing them to seek care outside their own region [27,28]. More broadly, studies on the subnational burden of disease have also confirmed that the southern and island regions perform worse in terms of life expectancy, healthy life expectancy and fatal burden, while the non-fatal burden increasingly affects older populations and women, further confirming that living longer does not necessarily mean living better [29].

Against this background, the shift from the demographic health dimension to the economic pension dimension is entirely coherent. A substantial body of the literature has shown that differential longevity is not only a demographic issue but also a distributive problem for pension systems [30,31,32]. In the Italian case, differences in mortality and survival by territory, gender and socio–economic position call into question the actuarial fairness of a system that, especially in its contributory component, uses coefficients based on average longevity values. Evidence on territorial differences in life expectancy at age 65 has shown that the South remains systematically below the Centre and especially the North-East, and that the use of uniform divisors tends to generate implicit mechanisms of taxation and subsidy across groups with different longevity profiles [33]. The same interpretive line had already emerged in earlier studies on the design of the Italian public pension system, which pointed out that survival differences by gender, cohort and area of residence could generate implicit redistributions not always consistent with the system’s principles of fairness [34,35]. Similarly, differences in longevity across older socio–economic groups suggest that the relationship between contributions paid and expected benefits is not socially neutral, with important implications for public pensions [36].

The assistance dimension likewise confirms the central role of territory. Economic and policy research has shown that the Italian long-term care system remains highly fragmented, with wide regional disparities in coverage, generosity of benefits and capacity to integrate health and social interventions [37]. Non-self-sufficient older people often have below-average economic resources and are therefore particularly exposed to the consequences of weak or fragmented public provision [38]. At the macro-institutional level, the regionalisation of healthcare and assistance has accentuated territorial differences in the ability to respond to need, while expenditure-containment policies have had non-negligible effects on avoidable mortality and interregional healthcare mobility, most sharply affecting the southern regions subjected to fiscal recovery plans [39,40]. Pensions and assistance, therefore, cannot be analysed separately from the demographic context: survival trajectories, the spread of disability and multimorbidity, and territorial capacity for care provision all jointly shape the pressure exerted by population ageing on the Italian welfare system. An approach that considers together the demographic conditions of survival and territorial differences in assistance benefits thus remains especially relevant because it is precisely at their intersection that the unequal regimes of ageing in Italy become fully visible.

## 3. Data and Methods

This study adopts a descriptive and territorially comparative approach to examine the relationship between demographic ageing, quality of survival and assistance benefits among older people in Italy. The analysis is based on aggregate data drawn from official statistical and institutional sources, primarily Istat, Eurostat and INPS, which provide information on the age structure of the population, life expectancy at birth (e_0_), life expectancy at age 65 (e_65_), healthy life expectancy at age 65 (e_65_^SLAQ^/e_65_^HLY^), severe limitations in daily activities, non-self-sufficiency and the distribution of assistance benefits among persons aged 65 and over.

The regional level constitutes the main unit of analysis, as it allows for demographic, health and welfare differences to be examined within a coherent territorial framework. Whenever permitted by the data source, the results are also presented by major geographical divisions, distinguishing between the north, the centre, and Mezzogiorno (which includes the regions Abruzzo, Molise, Campania, Apulia, Basilicata, and Calabria, as well as the two major islands, Sicily and Sardinia), and, for some health indicators, between the northwest, northeast, south, and islands. All variables are analysed separately by sex and, where possible, by age group, with particular attention to the categories of 65–74, 75–84, and 85 years and over.

To reconstruct the structural evolution of ageing, this study uses fixed-base relative growth indices, setting the older population observed on 1 January 1982 equal to 100 and comparing it with the population recorded on 1 January 2025. This is complemented by the ageing rate, defined as the population aged 65 and over per 100 inhabitants, and by the masculinity rate, measured as the number of men per 100 women. These indicators make it possible to capture both the intensity of demographic ageing and the changing gender composition of the older population.

The analysis of survival combines the quantity and quality of later life. Residual life expectancy at age 65 (e_65_) is used to measure the duration of survival, while healthy life expectancy at age 65 (e_65_^SLAQ^/e_65_^HLY^) captures the years expected to be lived without limitations in daily activities. Comparing these two indicators makes it possible to distinguish between the extension of life and the effective extension of life in good health. Because not all demographic, health and pension data are updated to the same reference year, and to ensure temporal comparability across indicators, average values from 2018 to 2023 are used whenever necessary. This choice also helps reduce the influence of short-term fluctuations linked to exceptional events, including the pandemic period.

Frailty is examined through rates of severe limitations in daily activities and, where available, indicators relating to multimorbidity and non-self-sufficiency. These measures are treated as proxies of health and functional vulnerability in old age. When the original source provides age-standardised rates, the analysis relies on those values to make regional comparisons more robust. The empirical strategy also includes exploratory bivariate comparisons, using national average values as graphical reference thresholds to identify territorial clusters and recurring configurations between ageing intensity, severe limitations and quality of survival. In addition, simple linear correlation coefficients are used to assess whether the regional intensity of demographic ageing is directly associated with the prevalence of severe limitations by sex.

The assistance dimension is analysed through the territorial distribution of assistance benefits, their aggregate amount, their incidence per 100 older people, and their breakdown by age and sex. In comparative terms, this study also considers regional rates of social security and assistance benefit receipt to highlight the uneven territorial weight of public support. Where specified by the source, these rates are interpreted in age-standardised form.

For clarity, five recurring concepts are used throughout the paper in the sense adopted by Italian official statistics. Frailty denotes an increased vulnerability to adverse health outcomes and is used here as an umbrella term; functional limitations refer to difficulties in performing basic activities of daily living without help, as measured by Istat/EHIS surveys; multimorbidity denotes the co-occurrence of two or more chronic conditions in the same individual; non-self-sufficiency (in Italian, non autosufficienza) refers to a condition requiring continuous assistance with essential daily activities and constitutes the main eligibility criterion for several Italian assistance benefits; assistance benefits (pensioni assistenziali) are non-contributory, means-tested and/or disability-based cash transfers funded through general taxation, distinct from contributory social-security pensions (pensioni previdenziali, pensioni contributive).

The purpose of the study is not to estimate causal effects but to identify empirical regularities and territorial and gender differentials linking longevity, frailty and reliance on public assistance. The methodological strategy is therefore explicitly descriptive and interpretative. It is intended to show that the pressure exerted by ageing on the Italian welfare system depends not only on the numerical growth of the older population, but also on the unequal territorial distribution of health conditions, functional limitations and assistance needs.

## 4. Results

### 4.1. The Demographic Structure of Population Ageing in Italy

The very rapid increase in the older population in Italy over the last 45 years (Table 1)—and, in particular, the growth of the oldest age group—took place against a very different backdrop for the total population: a phase of broad stagnation lasting until around the beginning of the 2000s, followed by a period of modest growth and, more recently, by a renewed slowdown—in some regions amounting to an actual decline—that has been especially marked in southern Italy.

**Figure 1:**
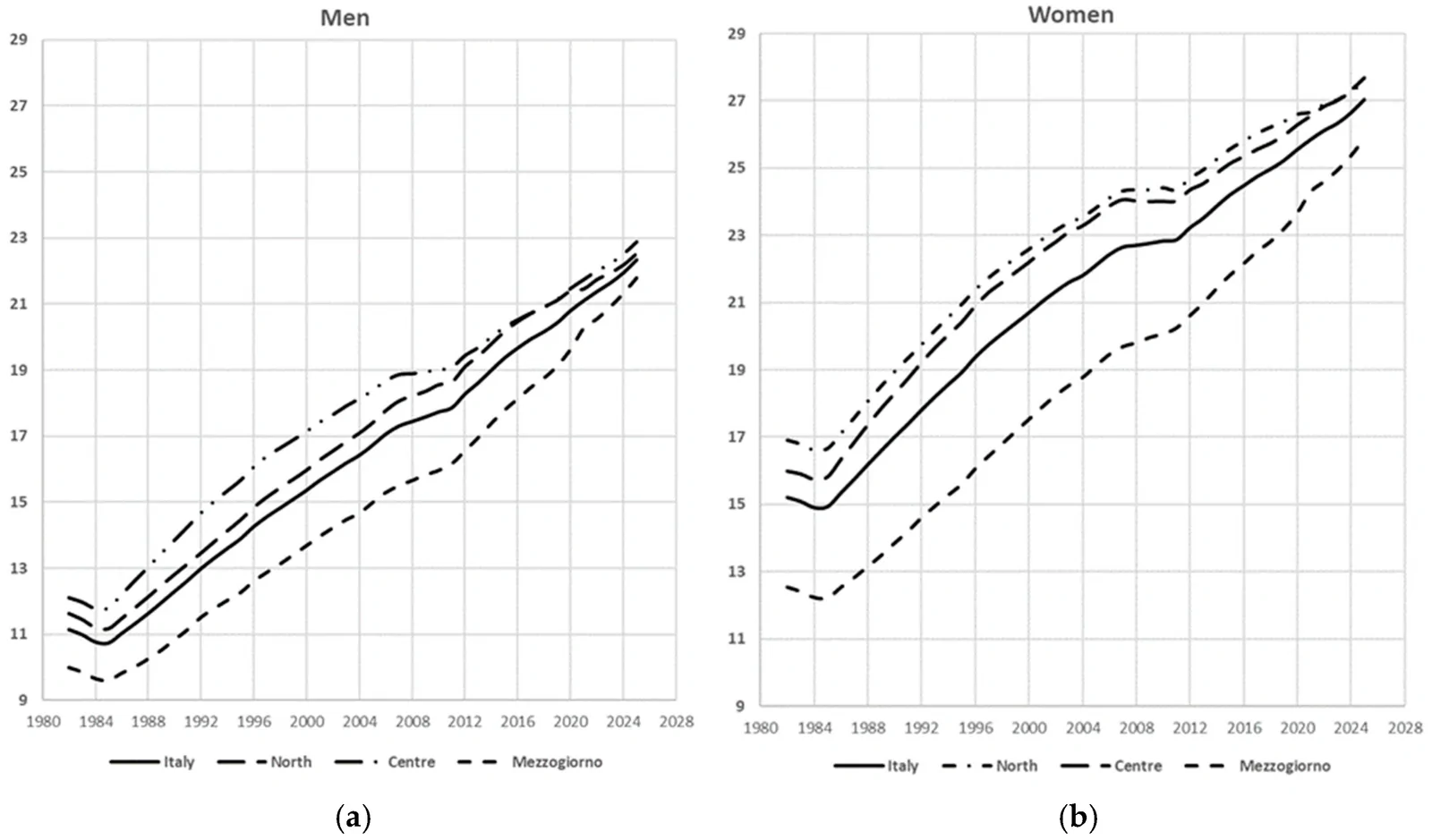
(**a**) Men and (**b**) women. Population ageing rates. Italy and major territorial divisions, 1982–2025. Source: Our own calculations based on Istat data.

The dynamics of the older population as a whole are quite different, however; it doubled over this period of time owing to the remarkable acceleration in the increase of those aged over 85 (Table 1), which became six times larger in the case of men and five times larger in the case of women. These latter developments took place in the context of two demographic subgroups that already had different levels of ageing by sex—comparatively higher in the case of women—and higher in the centre–north than in southern Italy, which was and still is the demographically “youngest” macro-region in Italy (Figure 1a,b). The growth in the rates of ageing showed a strong synchronicity in the north and in the centre, where the indices have settled at almost the same levels in recent years, while at the same time also recording a slight reduction. In southern Italy, on the other hand, although the rates long remained at markedly lower values, growth has been recorded in recent years, which, as Figure 1a,b again shows, has led them to converge towards the levels of the centre–north, especially in the case of men.

From the regional point of view, the process within these macro-areas has been characterised by strong variability, ranging between these two extremes: Liguria, which (2023) continues to be the region with the highest demographic ageing—the rate is close to 30%—and Campania, the one with the lowest (20%). As has already been seen (Table 1), the most substantial increase was recorded among those aged over 85, particularly men, a circumstance which, in the case of the north, contributed to rebalancing its structural profile by sex, which is currently more in line with the other two macro-areas than at the beginning of the 1980s (Table 2). Overall, this is the geographical division that, in relative terms, recorded the greatest increase in masculinity rates, while southern Italy recorded the most contained one (Table 3).

All these changes in the composition of the older population, which in the young-old age group are also the result of migration flows between macro-areas, derive more generally from the positive evolution of mortality, from which the last two age groups have benefited above all. It is thanks to these results that Italy ranks high among the countries with the highest human longevity in the EU. Indeed, if we consider the period 2018–2023, Italy was in fourth place for the number of years of life expectancy at birth (e_0_), both for men (80.8) and for women (85.2), surpassed in the first case, in order, by Switzerland, Norway and Sweden, and in the second case by Spain, Switzerland and France. Italy also retained the same position with regard to e_65_, the life expectancy at age 65 for women (22.3 years), while for men, the level of e_65_ (19.2 years) placed the country in seventh position, surpassed by Spain, France and Ireland in addition to Switzerland, Norway and Sweden. The male component presumably suffered more from the effects not only of the COVID-19 epidemic, but also of the heat waves and the very high incidence of influenza-like syndromes that have occurred, especially at the end of 2022 [41,42].

### 4.2. Health Conditions and Functional Limitations in Older Age

The lengthening of life expectancy at various ages is not, however, an indicator sensitive enough to highlight its quality, which is more closely linked to health status, but instead evaluates, schematically, only its duration. In the case of older people, it is therefore common practice to supplement the indication provided by e65 with further information about their condition in good health, which Eurostat specifies as follows: *a healthy condition is defined by the absence of limitations in functioning* (e_65_^HLY^); in Istat statistics, this indicator is referred to as “life expectancy free from limitations in daily activities” (e_65_^SLAQ^), from which the results of the progress made in improving the health conditions of the population may be grasped by assessing them in relation to the presence or absence of a wide range of functional limitations [43].

The examination of Eurostat data relating to e_65_^HLY^ (Table 4) already allows us to make an initial comparative reflection on the health conditions of the Italian case within the EU context. Indeed, it can be seen for both sexes, but particularly for women, that Italy falls in the compared with life expectancy at age 65, and these differences reveal that the ageing process, although common to all countries, is characterised in Italy by many heterogeneities of situation reflecting profound gender [44] and geographical gaps. Italy is among the countries where the durations of e_65_^HLY^ for men are generally higher than those for women, although at a very aggregated territorial level, such as the national one, the results for e_65_^HLY^ are the average from smaller areas: an example of this is the northeast (Table 4), where women’s e_65_^HLY^ was greater than that of men, albeit only slightly. Table 4 again clearly highlights the already well-known territorial inequalities of the country [45], which show a north–south health gradient through e_65_^HLY^ to the disadvantage of residents in the south, and which is particularly visible in the case of men (Table 4) and more generally for the regions of southern Italy. For many of the older people in southern Italy, social and health-related opportunities are comparatively lacking relative to the north. This can be seen in particular from the position of the northwest, which is placed within the subset of European countries that, judging by healthy life expectancy, health services, and living standards, contributes to building better quality ageing, which is reflected in the realisation of a more active role for older people in families and in society [46,47].

Regarding health conditions, Ref. [48] also provides rates of severe limitations in carrying out daily activities and offers a broad territorial disaggregation that makes it possible to integrate their results with those of e_65_^SLAQ^. Notably, these include indicators that make it possible to focus further on the picture of gender and territorial disparities in ageing experiences, insofar as such limitations form part of the set of vulnerabilities that define non-self-sufficiency (international classification of functioning, disability and health—ICF) [5].

Historical series for these rates (%) are available for 2008–2023, which so far show decreasing trends, although population ageing has increased (Figure 1); in recent years, they also display a certain convergence between macro-areas and between regions. For the purposes of the subsequent analyses, the averages relating to the period 2018–2023 are therefore considered, with reference to the two segments for which they are available: those aged 65 and over, consistent with the age group for which life expectancy e_65_^SLAQ^ (statistics provide life expectancy free from limitations in daily activities only for those aged 65 and over) is provided, and those aged 75 and over.

**Figure 2:**
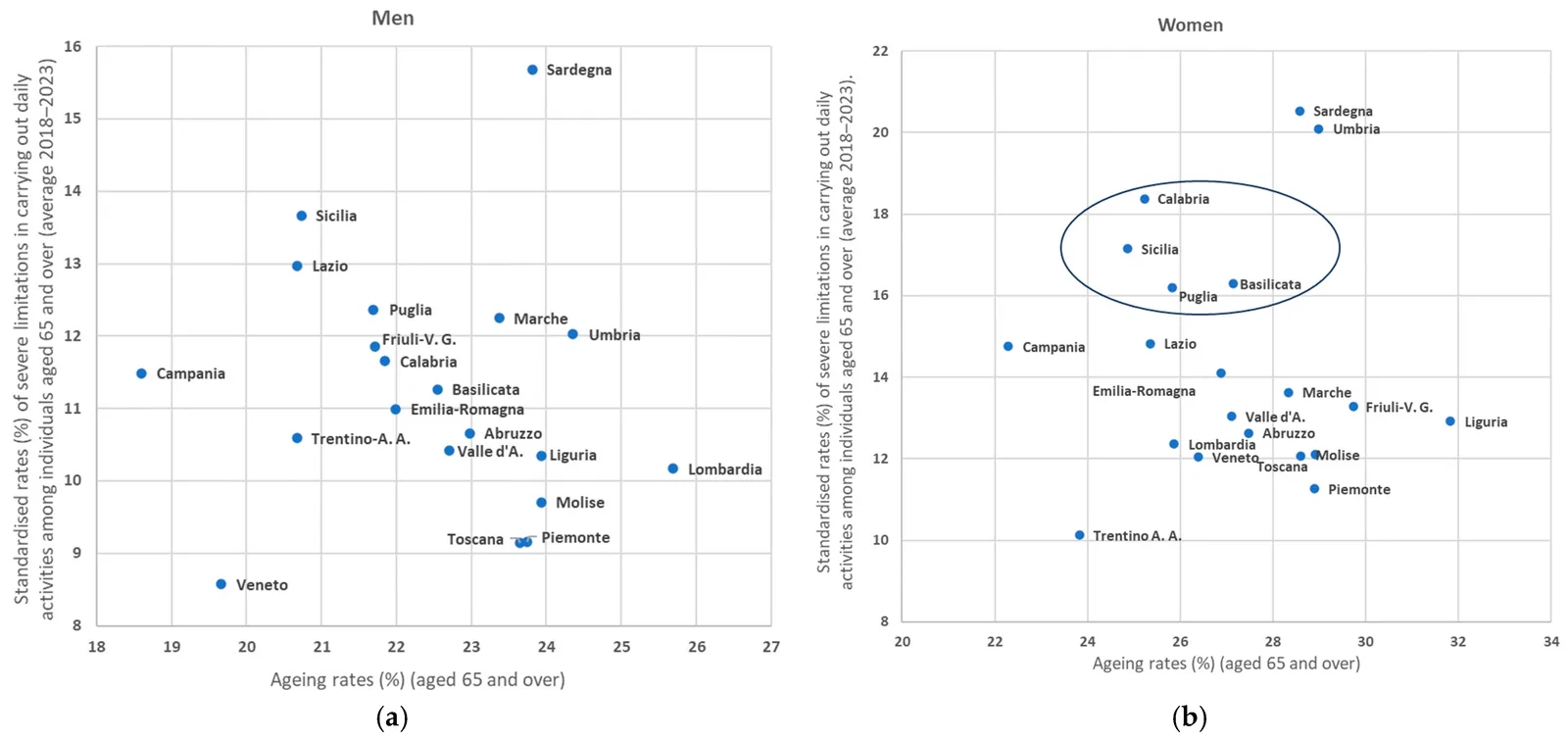
(**a**) Men and (**b**) women. Population ageing and severe limitations in carrying out daily activities. Average 2018–2023. Source: Our own calculations based on Istat data.

These data also show that the incidence of severe limitations (not only disability, but also non-self-sufficiency) is always higher among women than among men. In the comparison between regions (Figure 2a,b), the rate levels among women are, on average, 28% higher than men among those aged 65 and over, but with a maximum of 67% in Umbria, followed by Calabria with 58%. For those aged 75 and over, conditions are even worse for women, with rates on average being 48% higher, reaching a maximum of 89% in Veneto. These are significant differentials, considering that, for men, who display lower rates, these limitations may also be the consequence of road accidents or the after-effects of occupational diseases and/or workplace injuries [49], especially serious ones, events that are much less frequent among women.

**Figure 3:**
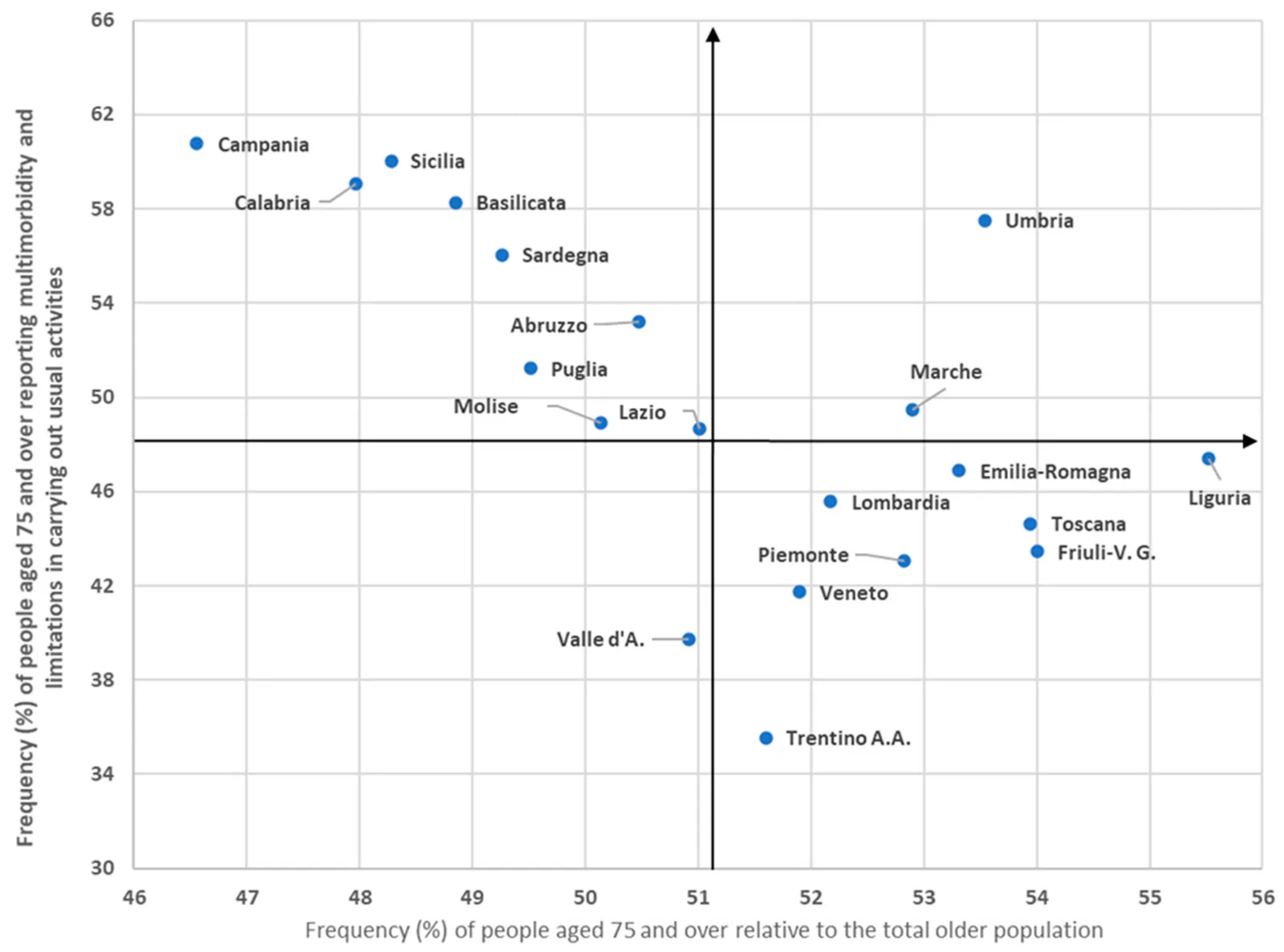
Frequency (%) of multimorbidity and severe limitations in usual activities among people aged 75 and over. Men and women (average 2018–2022). Source: Our own calculations based on Istat data. Note: At the national level, the frequency (%) of people aged 75 and over among the older population is 51.2%, while the proportion of those reporting multimorbidity and severe limitations in daily activities is 49.2%; this is highlighted by the two lines parallel to the Cartesian axes. These latter data are provided only for people aged 75 and over of both sexes and are currently available only up to 2022.

If we then consider the regional levels of population ageing (Figure 1a,b), together with the corresponding rates of severe limitations, again separately by sex (Figure 1b and Figure 2a,b), no interdependence or direct relationship is found, as indicated by the correlation coefficients between the two series of rates for both men (r = −0.1) and, to an even lesser degree, women (r = −0.04). What emerges instead is a strong variability in the two series of rates, especially for severe limitations; in the latter case, this variability denotes the presence of rather marked territorial differences. Indeed, from this point of view, Figure 2b anticipates the diversity among the regions of southern Italy for the rates of severe limitations, which are age-standardised: Abruzzo and Molise (located among the regions of the centre–north) can be noted, while Puglia, Basilicata, Sicily and Calabria constitute a separate subset when health conditions and ageing levels (65 years and over) are considered. Finally, the case of Sardinia is singular (Figure 2a,b), where, for both sexes, the rates of severe limitations are more than 40% higher than the national levels and are even higher for those aged 75 and over (Figure 3), where they reveal the widespread occurrence of much more critical health conditions.

The regional variability in the rates of severe limitations among those aged 65 and over therefore does not appear reducible solely to the different degree of demographic ageing. This is confirmed by the absence of a significant correlation between the two variables. Rather, this variation is linked to more complex structural factors, including socio–economic conditions, the quality and accessibility of health services, family care models, levels of prevention, and lifestyles. Territory thus appears to be not only a demographic context, but also an active determinant of health trajectories in old age.

Further examining the issue highlighted by these rates, it should be noted that if the share of non-self-sufficient persons among those aged over 65 at the national level was almost 13%, after age 75, this frequency increases by almost four times (49.2%), in part because of chronic diseases—that is, long-term pathologies (Figure 3). These are conditions that negatively affect quality of life and lead, in the advanced stages of old age, to significant increases in assistance needs that become ever more unavoidable, thus having inevitable repercussions on the need for care.

There are also strong territorial differences in the frequency of older people who find themselves in these more critical conditions. Indeed, the lines parallel to the Cartesian axes that in Figure 3 frame the location of the regions relative to the national context make it possible to identify the set of five regions of southern Italy already present in Figure 2a in the northeastern quadrant; they are recalled here because although there is a lower incidence of those aged over 75 there (see also Table 3), they present the highest frequency of non-self-sufficient persons. In the southwestern quadrant, we find the regions of the north, where the opposite occurs. There is thus a strong concentration of persons with serious difficulties in basic functions and worse health conditions in almost the whole of southern Italy, as well as in Umbria. This situation is plausibly attributable to poor prevention, to a demand for care that is not easily controllable and to different criteria in the assessment of health conditions, as will also be seen below.

It follows that the inequalities observed so far do not simply represent a quantitative imbalance in the distribution of the older population (Figure 2a,b and Figure 3), as may be inferred from the frequency of those aged 65 and over and those aged 75 and over, but rather a qualitative differentiation in the health conditions with which such older people face the more advanced ages. This aspect is particularly evident in southern Italy, where the higher incidence of frailty is accompanied by a more marked female exposure to non-self-sufficiency, with potential cumulative effects on the family and public care burden.

### 4.3. Longevity and Healthy Life Expectancy Across Regions

The reduction recorded between 2008 and 2023 in the frequencies of these pathologies was positively reflected in residual life expectancy at age 65, which, despite the turbulence of the COVID-19 period, was already almost at the same levels in 2023 as in 2019 throughout Italy. In particular, the growth of e_65_ exhibited a greater acceleration in the centre–north for both sexes, with the largest increases for men everywhere. However, the comparison regarding regional differences in e_65_ for both sexes in recent years highlights, on the one hand, very limited gaps among the regions of the north and the centre, including Abruzzo and Molise, as well as among the remaining regions of southern Italy. On the other hand, there is also a clear separation between these two subsets due to the fact that more situations of considerable disadvantage are recorded in these regions of southern Italy relative to those of the centre–north, a disadvantage that, in the comparison between Trentino-Alto Adige and Calabria and Sicily, reaches its highest value: one year and nine months (Table 5).

**Figure 4:**
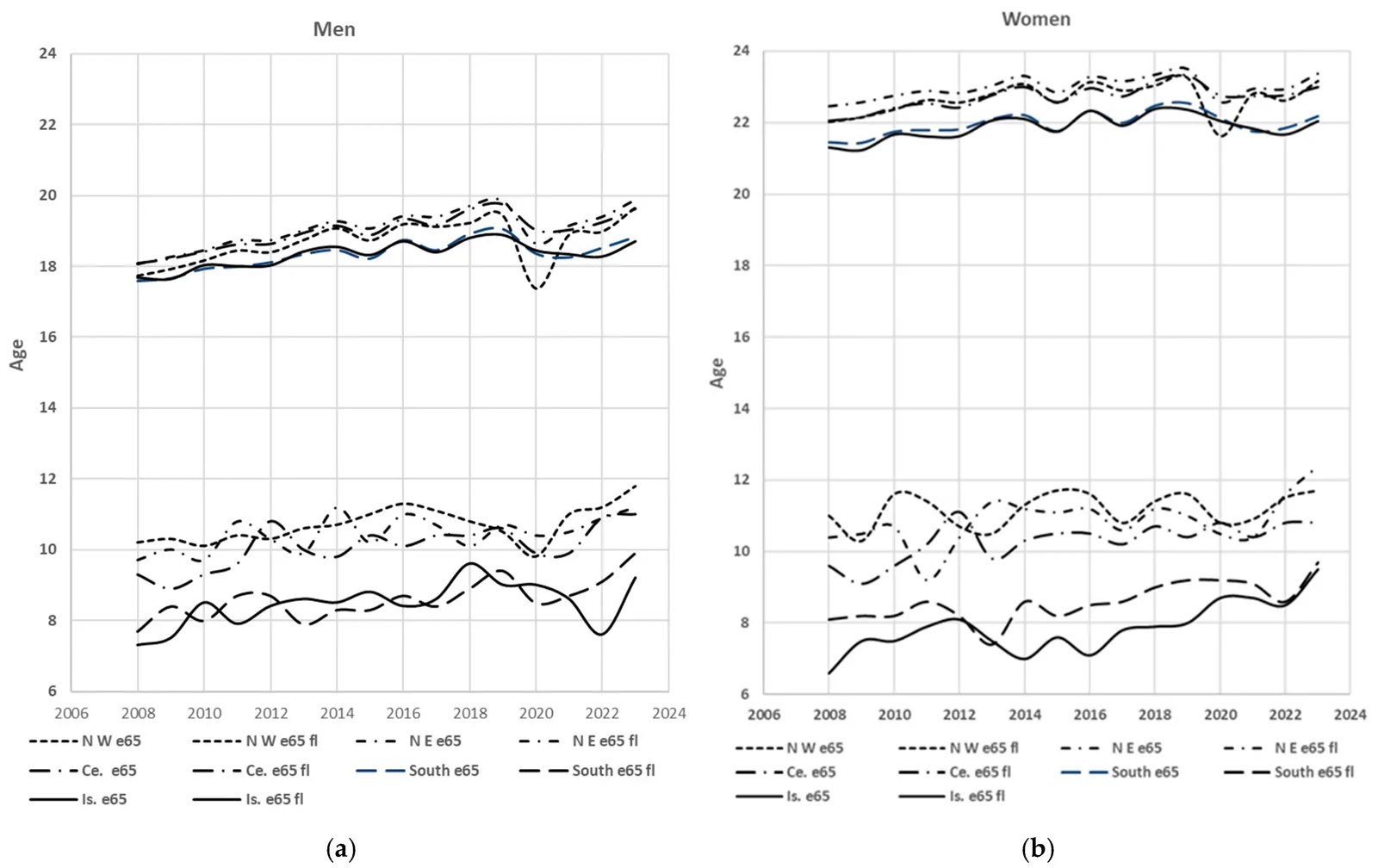
(**a**) Men and (**b**) women. Life expectancy at age 65 (e65) and life expectancy free from limitations (e65SLAQ), by sex. Geographical divisions. Averages 2008–2023. Source: Authors’ elaboration of Istat data.

Comparatively, the growth in life expectancy free from limitations in daily activities at age 65 (which is also an indirect indicator of absolute poverty) was greater than that of e^65^ in the centre and southern Italy for men and throughout Italy, particularly in the centre and southern Italy, for women. Despite this growth, the significant differences in terms of e_65_ between the southern regions and the rest of Italy (Table 5) are also found in the comparison of life expectancy free from limitations between the centre–north and southern Italy, where e_65_^SLAQ^ shows the lowest levels, with particularly low levels in the islands for women. This can be seen in Figure 4a,b, where the 2008–2023 dynamics of e_65_ (the trends farther from the horizontal axis) are compared with those of e_65_^SLAQ^ (closer to the horizontal axis) with reference to the five macro-areas. Again, the comparison between the profiles of the trends of e_65_ and e_65_^SLAQ^ for men and women highlights and anticipates what will be seen further on concerning the gender differences between e^65^ and e_65_^SLAQ^, which are not at all in harmony with one another because e_65_^SLAQ^ is directly influenced by the differentials in health conditions present at the territorial level. What emerges at this stage is the persistent disadvantage of southern Italy, where social inequalities in access to care would seem to penalise women more than men.

**Figure 5:**
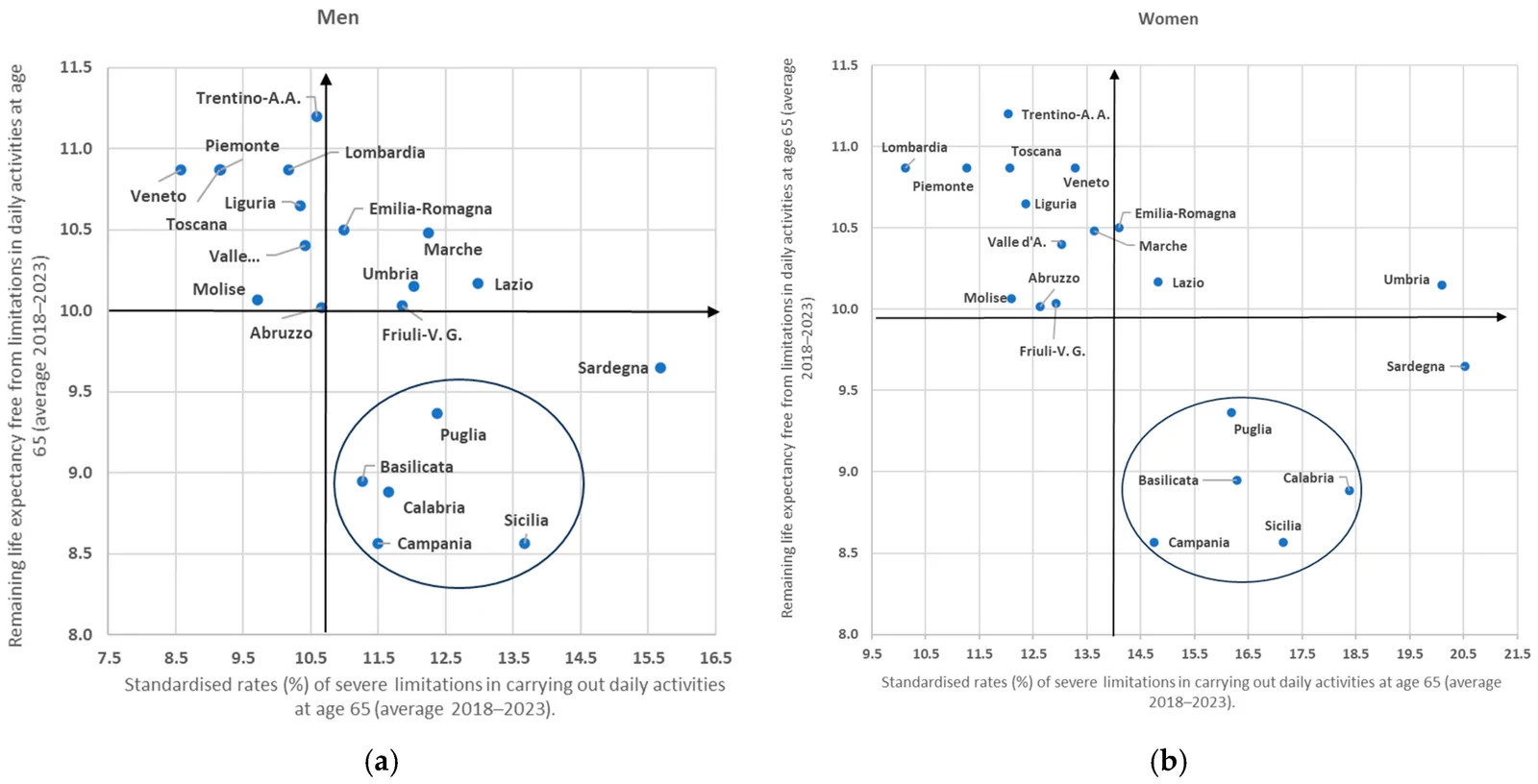
(**a**) Men and (**b**) women. Rates of severe limitations in daily activities and health conditions among individuals aged 65 and over. Source: Our own calculations based on Istat data. Note: At the national level, life expectancy free from limitations in daily activities at age 65 and over is 10.1 years for men and 9.9 years for women, as indicated by the lines parallel to the Cartesian axes.

A more general confirmation of this point emerges from the following figures (Figure 5a,b), where the rates of severe limitations in daily activities in each region are considered for both sexes and are associated with the corresponding e_65_^SLAQ^ of men and women. Here, the negative relationship between the two variables may be noted, and the lines parallel to the horizontal and vertical axes at the level of the national rates and e_65_^SLAQ^ again highlight that almost all the regions of southern Italy (except Abruzzo and Molise) are located in the southeastern quadrant—that is, the one where health inequalities are greatest. We again find the group already present in Figure 2b, along with Sardinia, always very isolated relative to the other regions and distinguished by the highest values for these rates. The geographical polarisation of the regions of the centre–north, together with Abruzzo and Molise, is once again emphasised, all of which differ from one another by only a few months on average, again in terms of e_65_^SLAQ^. There is also, however, the dispersion of Campania, Sicily, Puglia, Calabria and Sardinia facing a difficult situation due to the higher values for the severe limitation rates and the lower values for e_65_^SLAQ^. This is also graphic evidence fully in line with the picture of regional differences in e_65_ that had already emerged from Table 4. Both results were influenced by critical institutional and territorial factors, including a shortage of health personnel and the fragility of regional healthcare at different levels, with regions being precisely the territorial entities that plan and manage healthcare with full autonomy within their territorial scope [50]. From this point of view, gender differences and territorial inequalities take on a structural meaning because they directly affect the configuration of the demand for long-term care, modifying its intensity, duration and the necessary modes of response.

**Figure 6:**
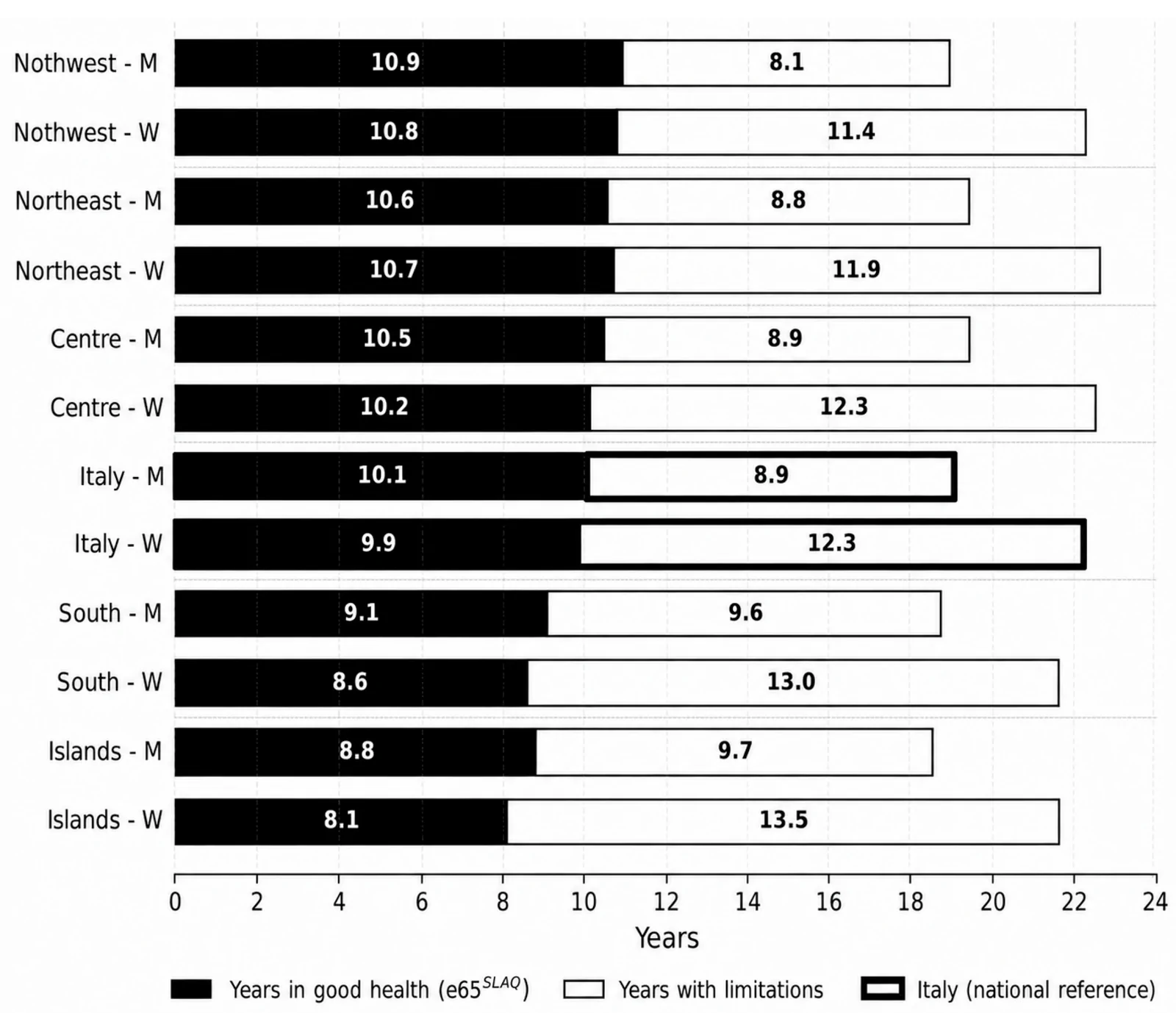
Remaining life expectancy at age 65 (average 2018–2023) in good health (black) and poor health (white), by sex (M and W) and territorial division. Years and decimals of a year. Source: Our own calculations based on Istat data.

The gender gap relating to e_65_ not only differs in terms of residual life between the sexes, which rather characterise gender differences within the country, but in terms of those that characterise residual life without limitations. Indeed, the gap between women’s e_65_ and e_65_^SLAQ^ relative to that of men continues to be wide in many cases, generally to the disadvantage of women [44]. In Italy, this occurs especially in the islands. However, more recently, this gap between e_65_ and e_65_^SLAQ^, usually in favour of men, is practically absent in the north; in the centre, it amounts only to a few months, while in the southern regions, it is on average more than one year. The result (Figure 6) is that women’s healthy life expectancy in the south and in the islands was and continues to be between 46% and 49% of e_65_ compared with an overall 54% or more in the centre–north.

This phenomenon is known as the gender health–survival paradox: women live longer than men [51,52] but, on average, they spend more years with chronic diseases and/or non-self-sufficiency [45]. In fact, the causes of men’s differential mortality at all ages are multiple—genetic factors, lifestyles and socio–economic factors—so that from each birth cohort far fewer men reach old age, but they tend to be healthier than women, who, as has been seen from the previous rates, tend to suffer more from long-term illnesses and disabling limitations, although they tend to experience fewer lethal illnesses than men.

The considerable gaps in the differences in e_65_^SLAQ^ highlighted by Figure 6, which denote the difficulties if not the impossibility of carrying out daily activities independently in the final phase of life, are therefore among the causes of the demand for assistance benefits. This is itself characterised by a gender and geographical gap. Territory thus appears not only as a demographic context but as an active factor in the differentiation of the trajectories of living conditions and health in old age.

### 4.4. Territorial Inequalities in Assistance Benefits

The protection of older people from poverty, and therefore also from poor health and social marginalisation (which are in close symbiosis), is a fundamental function of pension systems. The problem of social security is that among the requirements determining pension benefits, the continuity of employment remains key in addition to retirement age; there are corrective measures for assistance to older people when social security contributions that have been paid have been irregular or even absent, but even these envisage a broad profile of the assisted person. All of these socio–demographic “labels” cannot frame either the current or the past situation of the older person, insofar as not all people reach retirement in the same way. The result is therefore that the complexity of the different life courses is flattened. To this is added the fact that in Italy, the levels of pension treatment show strong disparities by sex as well as between private employees, public employees and self-employed workers [53,54], while assistance benefits are very modest [55]. This latter aspect is consistent with the persistent view of the family as the cornerstone of the living conditions of older people [56] and of the role of women as protagonists of underground welfare [57]. Indeed, at present, women in many cases have to reconcile their possible professional lives with family life. Other negative effects on women’s pension benefits must also be traced back to pay differences which, on average, are to their disadvantage; to a contribution seniority often shorter and more fragmented than that of men (again, for the reasons mentioned above); and to widowhood—on 1 January 2023, there was about one widower for every four widows—which, like all or in addition to the other elements, entails a reduced pension.

Within this framework of disparities, there is also the dualism of Italy’s economic ecosystems—again, the situation of southern Italy is notable, where the lesser presence of a pension culture connected with low levels of education leading to contribution evasion and avoidance, a greater diffusion of poor and/or undeclared work, and discontinuity of employment remain conditions that, within the current pension regulatory framework [4], erode the pension quantity or even lead to being unable to receive it—to the disadvantage of women in particular (i.e., the so-called pension gender gap) [58]. Health also obeys a rigid “social gradient” [59], which worsens progressively as one moves down the socio–economic scale. For those who are also in conditions of non-self-sufficiency, assistance benefits amounting to EUR 17.5 billion were provided in 2023, in addition to those strictly related to healthcare borne by the state and private parties. This is to say, tax and contribution exemptions, bonuses and concessions, and numerous assistance benefits not covered by social contributions but borne by general taxation are very widespread [4].

Within the category of assistance-type pensions in force at the beginning of 2023, 61% concerned older people, and this was divided as a social allowance among those (33.2%) who had insufficient income and/or inability to carry out normal daily functions (Figure 2) and as benefits for the disabled (for those lacking minimum income) among beneficiaries of benefits for former civilians with disabilities and deaf or mute people (66.8%), including the attendance allowance. The distribution by sex of all these benefits shows 33% were received by men and the remaining 67% was received by women [60]. The statistical documentation [60] makes it possible to examine in some detail the situation of recipients of pensions and social allowances because they are specifically those aged over 65, whereas disability protection has no lower age limit (as is instead the case for the previous assistance category). In any case, even in the case of disability, the percentage of benefits in favour of older people is just over 51%.

The evidence summarised in Table 6 highlights some of the features of the gradient discussed above. Older people in southern Italy account for almost 33% of the population compared with the rest of the country, but they receive more than 50% of both assistance pensions and their total amount (at the beginning of 2023, this amounted to EUR 5.391 billion). A common feature of these benefits is that their frequency is markedly higher among women than among men, and their incidence per 100 older people essentially doubles as one proceeds from the regions of the north to those of the south.

In light of what has been examined thus far, particularly regarding gender differences in the presence of severe limitations in functional activities (Figure 3), the provision of these benefits should inevitably reflect the imbalances in the structural characteristics of the older population by sex and age, as indeed is shown by Table 7. On average, women live longer than men (Figure 5), more frequently remain alone or in a condition of widowhood, and therefore with a reduced pension. By living longer, they also risk more frequently finding themselves in a precarious economic condition and with a greater risk of disability (Figure 6), so that, relative to men, women often come to receive more than one assistance benefit at the same time with increasing age (Table 7).

**Figure 7:**
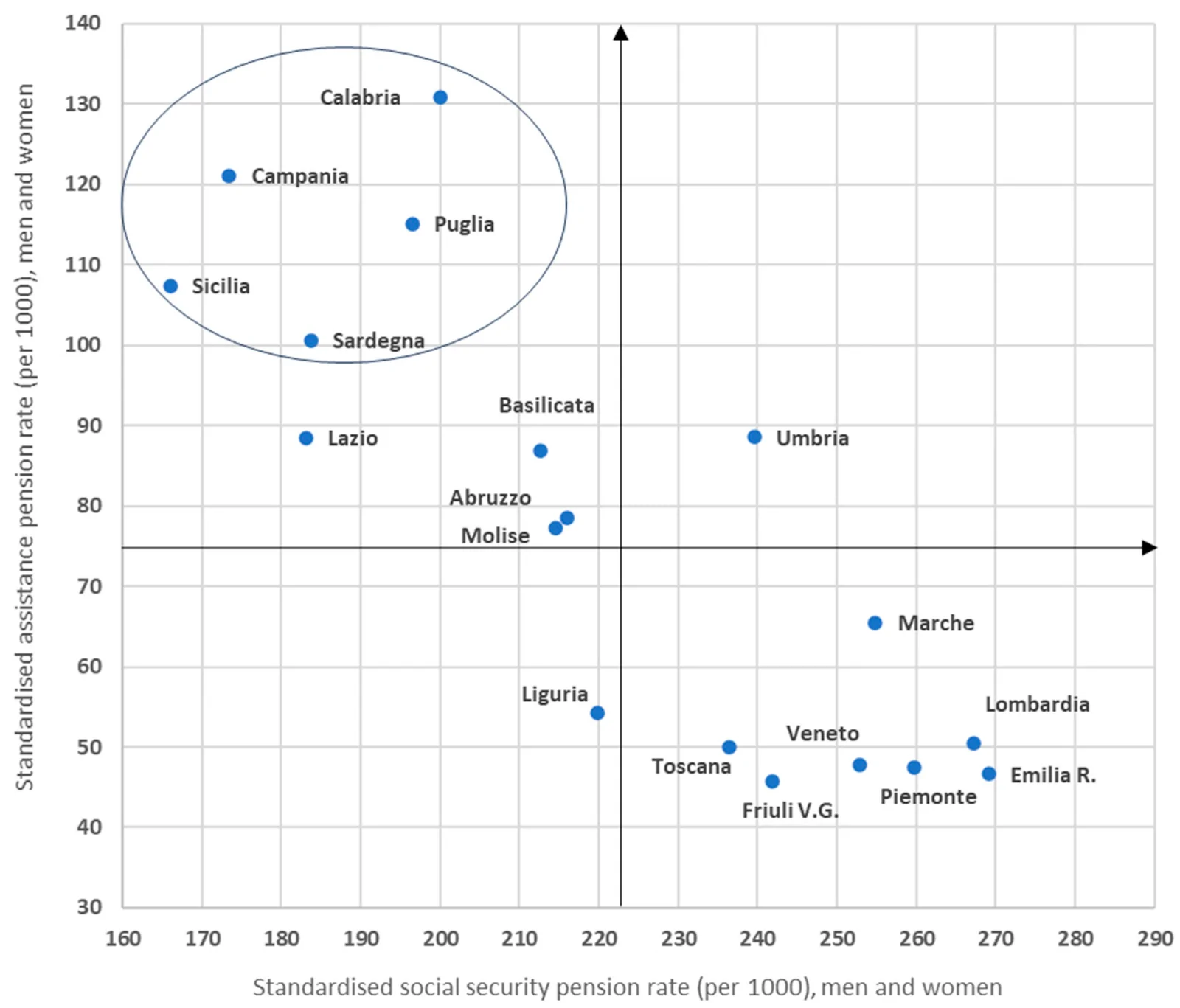
Standardised rates of social security and assistance benefits by region as of 1 January 2025. Sources: Our own calculations based on INPS^a^ data. Note: Social security pension rates refer to employees in the private sector. The graph does not include Trentino-Alto Adige and Valle d’Aosta because social allowances, insofar as they also constitute civil disability benefits, are paid directly by the autonomous provinces [6]. The two pension rates are calculated as the number of pensions per 1000 residents, standardised according to the age distribution of the population [60]. At the national level, the social security pension rate is 225.9, while the assistance benefit rate is 72.9; this is highlighted by the two lines parallel to the Cartesian axes.

These benefits are granted on the assumption of the existence of a health requirement and/or being below low-income thresholds, provided that an actual situation of need exists. These are situations that recur more frequently in the case of women, especially when they belong to older generations (Figure 7). From a socio–economic point of view, assistance benefits may therefore also perform a function of support to social security benefits, and this is what often occurs in southern Italy. Indeed, if we jointly consider the regional rates of social security and assistance benefit receipt, the same north–south dualism re-emerges that, in the previous figures, highlighted the differentials in the socio–health situations of the two macro-areas and is here illustrated by two extreme cases: on the one hand, Campania, with the lowest level of ageing and an assistance benefit rate only slightly lower than that of Calabria, which is the highest in absolute terms, and on the other hand, Liguria, the region with the highest level of ageing and an assistance benefit rate far lower than the previous ones. In addition to the low pension income from social security benefits [58], this assistance-based management of the conditions of older people in many areas of southern Italy is implicitly connected to the well-known shortage of services and provisions of the National Health Service in the south [50], thus, in fact, confirming the role of domestic care, which does not and cannot guarantee healthcare in a strict sense.

## 5. Discussion

As has appeared repeatedly in the results, there is a divide in the territory of Italy between areas with high levels of demographic ageing that do not correspond to proportional levels of disability and non-self-sufficiency and areas where the intensity of frailty among older individuals exceeds what would be expected based solely on age structure. It is precisely this mismatch between the quantitative and qualitative dimensions of ageing that places pressure on the welfare system. This pressure is not only due to the increase in the number of older individuals—although this has occurred (Table 1)—but more importantly to the health and functional composition of this population.

With respect to RQ.1, the analysis shows that demographic conditions and survival patterns among older individuals are not evenly distributed across the national territory. The centre–north exhibits, on average, more favourable levels of longevity and survival quality, whereas the south and the islands show a higher concentration of frailty, functional limitations and non-self-sufficiency. This results in a geography of unequal ageing, where the length of life and the conditions under which it is extended do not necessarily coincide. This pattern is particularly evident among women, who continue to experience a more pronounced gap between longevity and health.

The need for assistance for frail older individuals is supported through a mixed model in Italy, including monetary transfers (i.e., welfare assistance), healthcare provision and informal family care. As already noted, territorial disparities in healthcare provision are well documented [50], to the disadvantage of the south and the islands. At the same time, the prospects for family solidarity are increasingly undermined by changes in family structures and the weakening of kinship networks. The continuity of such solidarity—still largely considered a cornerstone of care provision—is becoming progressively less compatible with the spread of non-marital cohabitation, increasing marital instability and divorce rates, shrinking family size, and broader demographic dynamics. If a hypothetical caregiver is assumed to be in the 50–70 age group, it is worth noting that while there were on average 2.5 women per individual aged 75 and over in the early 1980s, when population ageing began to intensify, this ratio has now almost halved to 1.3 and is expected to decline further.

With reference to RQ.2, the results clearly show that the distribution and intensity of assistance benefits do not simply follow the numerical weight of the older population but instead reflect deep territorial and gender inequalities. The Mezzogiorno, despite hosting a smaller share of older individuals compared to the centre–north, absorbs a significantly larger share of assistance benefits in terms of both the number of recipients and the amount they receive, with incidence levels per 100 older individuals being markedly higher than those observed in the north. This imbalance is even more pronounced among women, further confirming that greater health and socio–economic vulnerability translate into stronger dependence on public support.

These transformations put both the continuity and the characteristics of family support and responsibilities at risk, as is also evidenced by the transfer of caregiving duties to younger household members when families are unable to afford external care. This phenomenon is more widespread in the south, and Istat [61] reported in its latest update of the survey on aspects of daily life that 7% of young people aged 15–24 in Italy were engaged in caring for frail adults or older individuals, with inevitable negative consequences for their education and labour market integration.

In the context of the weakening of family networks, and particularly for older individuals not living within a family environment, alternative responses are provided by public welfare services. These include integrated home care, with services also delivered by healthcare professionals, and residential socio–health and socio–assistance facilities, where, according to the latest Istat survey (Table 1), more than 75% of residents were aged 65 and over. However, significant territorial imbalances persist, not only in the distribution of facilities but also in the availability of beds. Per capita availability remains approximately halved from the north to the centre and reduced to less than one-third in the south, as highlighted by Istat surveys conducted at the end of 2013 and 2022.

Future prospects (Table 8) for care needs among older individuals remain highly uncertain. On the one hand, many health conditions associated with ageing originate earlier in life but manifest later. On the other hand, profound socio–cultural changes are reshaping the ageing process, which is no longer framed solely as “old age” but as a new phase of life, supported by advances in healthy longevity. These developments are driven by interdisciplinary research in medicine, pharmacology, biology and technological innovation, which provide new strategies in the field of health. In this context, new multifunctional housing solutions have emerged that offer alternative living arrangements aligned with the concept of healthy longevity and primarily target younger cohorts of older individuals, often within the private sector.

These trends can also be interpreted as an extension of the so-called silver economy [47], whose expansion is expected to be reinforced by the entry into old age of the last baby boomer cohorts—those born between 1960 and 1967—who are numerically larger than previous generations and are expected, on average, to age with better resources and performance levels, higher educational attainment, and improved living standards [62]. However, a major uncertainty concerns the impact of this generational transition on the already marked socio–economic and cultural heterogeneity of the older population [63], as well as the fact that healthy longevity is not territorially neutral. The risk is that Italy will not age uniformly but will instead develop distinct territorial regimes of longevity and fragility.

## 6. Conclusions and Policy Implications

This study has shown that population ageing in Italy cannot be interpreted as a purely demographic phenomenon, but rather as the outcome of a complex interaction between survival dynamics, health conditions and welfare arrangements, all of which are deeply embedded in territorial contexts. The evidence confirms that the Italian case is characterised by a persistent and structured heterogeneity, in which regional disparities in health, access to care, and socio–economic conditions shape distinct trajectories of ageing. In line with the broader literature on health inequalities and survival [59], the results reinforce the idea that longevity gains, while substantial, do not automatically translate into improvements in the quality of life at older ages. On the contrary, they may coexist with significant levels of frailty, multimorbidity and functional limitations, especially in contexts where access to healthcare and preventive services is uneven. The Italian case thus exemplifies how the extension of life expectancy, particularly at older ages, may amplify rather than reduce existing inequalities when the distribution of health resources remains unbalanced.

The findings also highlight the central role of territory as an active determinant of ageing processes. Regional healthcare systems, which operate with a high degree of autonomy, help to shape differentiated outcomes in both survival and care provision [50]. As a result, the well-known north–south divide continues to appear not only in economic terms but also in the configuration of health conditions and access to long-term care services, thus confirming earlier evidence on the persistence—and in some cases the widening—of territorial inequalities in Italy [18,19]. From a welfare perspective, the results suggest that the current Italian model—based on a combination of monetary transfers, healthcare provision and informal family care—is increasingly under strain. While public expenditure on assistance remains substantial (and reaches significant levels in relation to GDP), the system continues to rely heavily on family-based care, particularly in southern regions where formal services are less developed. However, as widely documented in the literature on welfare regimes [53,57], this model appears to be progressively less sustainable in the face of ongoing demographic and social transformations, including population ageing, declining fertility and changing family structures.

At the same time, the persistence of gender inequalities in health and care needs—already well documented in demographic and epidemiological research [45,51]—emerges as a critical dimension of the ageing process. Women’s longer survival, combined with a higher prevalence of disability and non-self-sufficiency, translates into a disproportionate exposure to care dependency and a greater reliance on both informal and formal support systems. This confirms the structural nature of the so-called health–survival paradox and its implications for the organisation of long-term care.

Looking ahead, the transition of the baby boomer cohorts into older age is likely to further reshape the landscape of ageing in Italy. On the one hand, these cohorts are expected to reach older ages with better educational levels, economic resources and health conditions [62]. On the other hand, their entry into the later stages of life may amplify existing socio–economic and territorial inequalities, particularly if access to the benefits of healthy longevity remains uneven [63]. The expansion of the silver economy [47] and the emergence of new housing and care models oriented towards active and healthy ageing represent important developments, but they also risk increasing segmentation between more and less advantaged groups of older individuals.

This study has some limitations that should be acknowledged. First, the analysis is explicitly descriptive and correlational: it identifies territorial and gender regularities but does not estimate causal relationships between longevity, health status and access to assistance. Second, the study relies on secondary aggregate data from Istat, Eurostat and INPS, collected for administrative and statistical purposes rather than designed specifically to answer the research questions addressed here; this also explains the heterogeneous reference periods across indicators (1 January 2025 for demographic-structure data, 2018–2023 averages for health and disability indicators, and either 2023 or 2025 for assistance-benefit data, depending on data availability). Third, the regional level of analysis, while informative, may mask relevant intra-regional heterogeneity at the provincial or municipal level. Fourth, several health indicators are based on self-reported measures (e.g., EHIS-based severe limitations in daily activities), which may be subject to cross-regional differences in reporting behaviour and cultural perceptions of health and disability. Future research should therefore complement this descriptive framework with multivariate or multilevel models capable of controlling for socio–economic confounders, exploit linked administrative microdata to move beyond aggregate indicators, monitor the post-pandemic evolution of the trends documented here, and extend the comparative perspective to other Southern European welfare states facing similar patterns of population ageing and territorial inequality.

From a policy standpoint, these considerations call for a rethinking of ageing policies along three main directions. First, there is a clear need to strengthen preventive strategies across the life course, in line with the long-standing evidence that many health conditions in old age originate earlier in life [64]. Second, reducing territorial inequalities requires a more balanced allocation of healthcare resources and a reinforcement of service provision in disadvantaged regions, particularly in the field of long-term care, where shortages in infrastructure and personnel remain significant [65,66]. Third, the progressive weakening of informal care networks makes it necessary to develop more robust and integrated systems of formal care that are capable of complementing rather than substituting family support. Ultimately, the Italian case underscores the importance of adopting a multidimensional and territorially grounded approach to ageing, capable of integrating demographic, health and welfare perspectives. Only by recognising the heterogeneity of ageing trajectories and addressing the structural inequalities that underpin them will it be possible to design policies that ensure not only longer lives, but also more equitable and sustainable conditions in later life.

## Figures and Tables

**Table 1:** Relative increase in the older population by geographical division and sex as of 1 January 2025. Index number = 100 on 1 January 1982.

Age	North	Centre	Mezzogiorno	Italy
		Men		
65–74	154.2	151.5	170.4	158.8
75–84	280.5	267.2	262.2	271.7
85 and over	691.4	652.8	560.5	639.3
65 and over	210.9	205.5	213.7	210.7
		Women		
65–74	124.9	136.6	158.7	137.3
75–84	189.0	209.6	228.7	204.2
85 and over	491.5	502.2	496.3	495.1
65 and over	171.8	186.9	203.9	184.2

Source: Authors’ elaboration of Istat data.

**Table 2:** Masculinity rates (men per 100 women) in the older population by geographical division. 1 January 1982 and 1 January 2025.

Age				
	65–74	75–84	85 e+	65 e+
Italy				
1982	77.7	59.5	42.4	69.4
2025	89.9	79.2	54.8	79.4
North				
1982	73.4	53.3	38.0	64.3
2025	90.6	79.1	53.4	78.9
Centre				
1982	79.9	61.4	42.7	71.3
2025	88.6	78.3	55.5	78.3
Mezzogiorno				
1982	83.7	69.6	50.0	77.0
2025	89.8	79.8	56.5	80.6

Source: Authors’ elaboration of Istat data.

**Table 3:** Structure (%) of the older population by age, sex and geographical division; 1 January 1982 and 1 January 2025.

		Men				Women	
Age	1982		2025		1982		2025
Italy							
65–74	68.2		51.4		60.9		45.4
75–84	27.4		35.4		32.0		35.4
85 and over	4.4		13.2		7.1		19.2
Total	100.0		100.0		100.0		100.0
North							
65–74	68.7		50.3		60.2		43.8
75–84	27.1		36.0		32.7		36.0
85 and over	4.2		13.7		7.1		20.2
Total	100.0		100.0		100.0		100.0
Centre							
65–74	68.2		50.3		60.8		44.5
75–84	27.4		35.6		31.8		35.6
85 and over	4.4		14.1		7.4		20.9
Total	100.0		100.0		100.0		100.0
Mezzogiorno							
65–74	67.6		53.9		62.2		48.4
75–84	27.9		34.2		30.8		34.6
85 and over	4.5		11.9		7.0		17.0
Total	100.0		100.0		100.0		100.0

Source: Authors’ elaboration of Istat data.

**Table 4:** Healthy life expectancy (e_65_^HLY^) by country, sex, and, for Italy, by geographical division (*) (averages 2018–2023).

Country	Men	Country	Women
Norway	14.98	Sweden	15.37
Sweden	14.73	Norway	14.96
Malta	13.08	Malta	13.03
Ireland	11.93	Ireland	12.87
Spain	11.05	France	11.83
Switzerland	11.02	Belgium	11.15
* Northwest (IT)	10.85	Denmark	11.07
Belgium	10.82	Germany	10.95
* Northeast (IT)	10.63	Spain	10.85
France	10.47	* Northwest (IT)	10.82
* Centre (IT)	10.45	* Northeast (IT)	10.68
Denmark	10.15	Switzerland	10.50
Italy	10.10	Slovenia	10.50
Germany	10.03	Bulgaria	10.40
Netherlands	9.63	* Centre (IT)	10.15
Slovenia	9.47	Finland	9.92
Finland	9.43	Italy	9.88
* South (IT)	9.08	Netherlands	9.42
Bulgaria	8.98	Poland	8.85
* Islands (IT)	8.83	Austria	8.67
Austria	8.53	* South (IT)	8.63
Cyprus	8.37	* Islands (IT)	8.05
Portugal	8.37	Czechia	8.05

Source: Authors’ elaboration of Istat data and European Health Interview Survey (EHIS). Note: Not all the available demographic data relating to health conditions and pension benefits are updated to 2025; where necessary and to maintain the necessary homogeneity, reference is therefore made in this table and in the following pages to the period 2018–2023, for which the average values are provided. Note: (*) the geographical divisions refer to the Italian macro-areas.

**Table 5:** Differences between the residual life expectancy at age 65 of each region and that of all the others. Men and women, averages 2018–2023.

	Piemonte	Valle d’Aosta	Liguria	Lombardia	Trentino A.A.	Veneto	Friuli-V.G.	Emilia-R.	Toscana	Umbria	Marche	Lazio	Abruzzo	Molise	Campania	Puglia	Basilicata	Calabria	Sicilia	Sardegna
Piemonte	0.00																			
Valle d’A.	0.47	0.00																		
Liguria	0.22	0.25	0.00																	
Lombardia	0.00	0.47	0.22	0.00																
Trenti.A.A.	0.33	0.80	0.55	0.33	0.00															
Veneto	0.00	0.47	0.22	0.00	0.33	0.00														
Friuli-V.G.	0.83	0.37	0.62	0.83	1.17	0.83	0.00													
Emilia-R.	0.37	0.10	0.15	0.37	0.70	0.37	0.47	0.00												
Toscana	0.00	0.47	0.22	0.00	0.33	0.00	0.83	0.37	0.00											
Umbria	0.72	0.25	0.50	0.72	1.05	0.72	0.12	0.35	0.72	0.00										
Marche	0.38	0.08	0.17	0.38	0.72	0.38	0.45	0.02	0.38	0.33	0.00									
Lazio	0.70	0.23	0.48	0.70	1.03	0.70	0.13	0.33	0.70	0.02	0.32	0.00								
Abruzzo	0.85	0.38	0.63	0.85	1.18	0.85	0.02	0.48	0.85	0.13	0.47	0.15	0.00							
Molise	0.80	0.33	0.58	0.80	1.13	0.80	0.03	0.43	0.80	0.08	0.42	0.10	0.05	0.00						
Campania	2.30	1.83	2.08	2.30	2.63	2.30	1.47	1.93	2.30	1.58	1.92	1.60	1.45	1.50	0.00					
Puglia	1.50	1.03	1.28	1.50	1.83	1.50	0.67	1.13	1.50	0.78	1.12	0.80	0.65	0.70	0.80	0.00				
Basilicata	1.92	1.45	1.70	1.92	2.25	1.92	1.08	1.55	1.92	1.20	1.53	1.22	1.07	1.12	0.38	0.42	0.00			
Calabria	1.98	1.52	1.77	1.98	2.32	1.98	1.15	1.62	1.98	1.27	1.60	1.28	1.13	1.18	0.32	0.48	0.07	0.00		
Sicilia	2.30	1.83	2.08	2.30	2.63	2.30	1.47	1.93	2.30	1.58	1.92	1.60	1.45	1.50	0.00	0.80	0.38	0.32	0.00	
Sardegna	1.22	0.75	1.00	1.22	1.55	1.22	0.38	0.85	1.22	0.50	0.83	0.52	0.37	0.42	1.08	0.28	0.70	0.77	1.08	0.00

Source: Authors’ elaboration of Istat data.

**Table 6:** Distribution (%) of the older population and pensions as of 1 January 2023, by gender and territory.

Macro-Regions	Men (a)	Men (b)	Men (c)	Men (d)	Women (a)	Women (b)	Women (c)	Women (d)
North	47.2	22.8	21.4	2.2	47.6	25.1	24.6	3.3
Centre	20.3	18.5	17.6	4.3	20.5	21.1	20.8	6.5
Mezzogiorno	32.5	58.7	61.1	9.2	31.9	53.8	54.6	11.0

Note: (a) Territorial distribution of the older population, %. (b) Distribution of the total pension amount, %. (c) Territorial distribution of the number of pensions, %. (d) Number of pensions per 100 older persons of the same sex. Source: Authors’ elaboration of Istat and INPS^a^ data.

**Table 7:** Assistance benefits in force as of 1 January 2025 relating to older adults residing in Italy aged 65 years and over; percentages by age and sex.

Age Group	Men (a)	Men (b)	Men (c)	M. Rate (**)	Women (a)	Women (b)	Women (c)
65 to 69	19.1	27.8	9.5	91.5	12.7	24.1	11.4
70 to 74	19.5	23.7	11.3	88.2	13.7	21.3	13.8
75 to 79	18.0	21.1	11.7	83.3	14.9	20.1	16.0
80 to 84	15.5	14.3	14.9	73.8	16.3	15.4	22.8
85 to 89	15.3	9.2	22.8	62.7	20.0	11.7	36.9
90 and over	12.6	4.0	43.2	43.5	22.4	7.3	66.0
Overall	100.0	100.0	13.7	79.5	100.0	100.0	21.6

Note: (a) Structure of assistance benefits (%). (b) Structure of the older population (%). (c) Assistance benefits per 100 older people of the same sex. (**) Number of men per 100 women. Source: Authors’ elaboration of Istat and INPS^a^ data.

**Table 8:** Beds in residential socio–health and socio–assistance facilities for older guests, by geographical division. 31 December 2013 and 2022, rates per 1000 residents.

Prospect Date	Northwest	Northeast	Centre	South	Islands	Italy
31 December 2013	31.5	30.6	14.7	9.5	13.8	21.4
31 December 2022	28.8	29.1	13.8	8.4	9.1	19.3

Source: Authors’ elaboration of Istat data (2015 and 2025).

## Data Availability

Data were derived from publicly available sources listed in the References.
